# Bis{2-meth­oxy-6-[(*E*)-(4-methyl­benz­yl)imino­meth­yl]phenolato}palladium(II) chloro­form monosolvate

**DOI:** 10.1107/S1600536814015025

**Published:** 2014-07-02

**Authors:** Hadariah Bahron, Amalina Mohd Tajuddin, Wan Nazihah Wan Ibrahim, Suchada Chantrapromma, Hoong-Kun Fun

**Affiliations:** a Faculty of Applied Sciences, Universiti Teknologi MARA, 40450 Shah Alam, Selangor, Malaysia; b DDH CoRe, Universiti Teknologi MARA, 40450 Shah Alam, Selangor, Malaysia; c Department of Chemistry, Faculty of Science, Prince of Songkla University, Hat-Yai, Songkhla 90112, Thailand; d X-ray Crystallography Unit, School of Physics, Universiti Sains Malaysia, 11800 USM, Penang, Malaysia; e Department of Pharmaceutical Chemistry, College of Pharmacy, King Saud University, PO Box 2457, Riyadh 11451, Saudi Arabia

**Keywords:** crystal structure

## Abstract

In the title complex, [Pd(C_16_H_16_NO_2_)_2_]·CHCl_3_, the Pd^II^ cation lies on an inversion center. One Cl atom of the CHCl_3_ solvent mol­ecule lies on a twofold axis and the C—H group is disordered with equal occupancies about this axis with the other Cl atom in a general position with full occupancy. The Pd^II^ cation is four-coordinate and adopts a square-planar geometry *via* coordination of the imine N and phenolic O atoms of the two bidentate Schiff base anions. The N and O atoms of these ligands are mutually *trans*. The plane of the benzene ring makes a dihedral angle of 73.52 (10)° with that of the meth­oxy­phenolate ring. In the crystal, mol­ecules of the Pd^II^ complex are arranged into sheets parallel to the *ac* plane, and the chloro­form solvent mol­ecules are located in the inter­stitial areas between the complex mol­ecules. Weak inter­molecular C—H⋯O and C—H⋯π inter­actions stabilize the packing.

## Related literature   

For bond-length data, see: Allen *et al.* (1987 ▶). For related structures, see: Bahron *et al.* (2011**a* ▶,b* ▶); Halder *et al.* (2008 ▶). For background to and applications of Pd^II^ complexes, see: Bowes *et al.* (2011 ▶); Geeta *et al.* (2010 ▶); Gupta & Sutar (2008 ▶); Kalita *et al.* (2014 ▶); Mohd Tajuddin *et al.* (2012 ▶); Tamizh & Karvembu (2012 ▶).
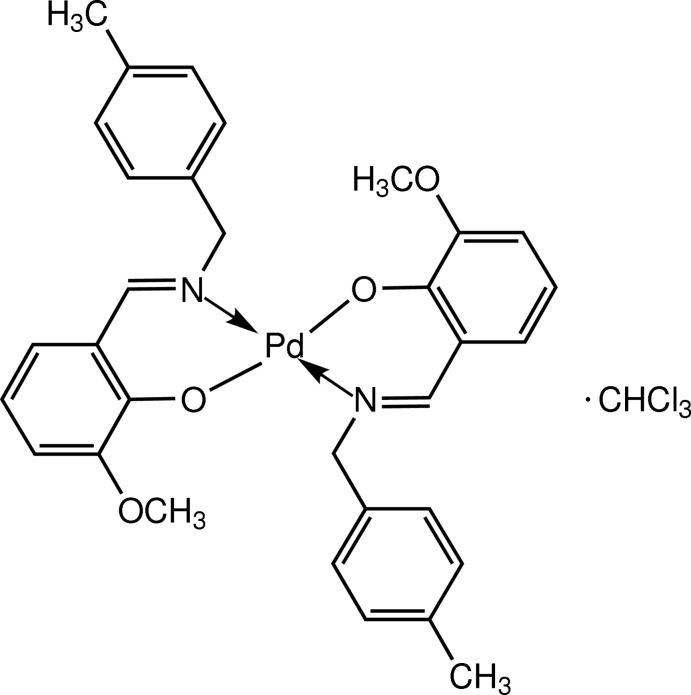


## Experimental   

### 

#### Crystal data  

[Pd(C_16_H_16_NO_2_)_2_]·CHCl_3_
*M* *_r_* = 734.36
Monoclinic, 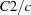

*a* = 31.9861 (8) Å
*b* = 5.9668 (2) Å
*c* = 22.6135 (5) Å
β = 134.885 (1)°
*V* = 3057.92 (15) Å^3^
*Z* = 4
Mo *K*α radiation
μ = 0.91 mm^−1^
*T* = 100 K
0.48 × 0.25 × 0.18 mm

#### Data collection  

Bruker APEXII CCD area-detector diffractometer
Absorption correction: multi-scan (*SADABS*; Bruker, 2009 ▶) *T*_min_ = 0.669, *T*_max_ = 0.853
43800 measured reflections
5542 independent reflections
5006 reflections with *I* > 2σ(*I*)
*R*_int_ = 0.021

#### Refinement  

*R*[*F* ^2^ > 2σ(*F* ^2^)] = 0.029
*wR*(*F* ^2^) = 0.073
*S* = 1.05
5542 reflections
204 parameters
H-atom parameters constrained
Δρ_max_ = 1.24 e Å^−3^
Δρ_min_ = −1.90 e Å^−3^

### 

Data collection: *APEX2* (Bruker, 2009 ▶); cell refinement: *SAINT* (Bruker, 2009 ▶); data reduction: *SAINT*; program(s) used to solve structure: *SHELXTL* (Sheldrick, 2008 ▶); program(s) used to refine structure: *SHELXTL*; molecular graphics: *SHELXTL*; software used to prepare material for publication: *SHELXTL*, *PLATON* (Spek, 2009 ▶) and *publCIF* (Westrip, 2010 ▶).

## Supplementary Material

Crystal structure: contains datablock(s) global, I. DOI: 10.1107/S1600536814015025/sj5416sup1.cif

Structure factors: contains datablock(s) I. DOI: 10.1107/S1600536814015025/sj5416Isup2.hkl

CCDC reference: 1010352

Additional supporting information:  crystallographic information; 3D view; checkCIF report

## Figures and Tables

**Table 1:** Hydrogen-bond geometry (Å, °) *Cg*1 is the centroid of the C9–C14 ring.

*D*—H⋯*A*	*D*—H	H⋯*A*	*D*⋯*A*	*D*—H⋯*A*
C8—H8*A*⋯O1^i^	0.97	2.19	2.806 (2)	120
C14—H14*A*⋯O1^i^	0.93	2.57	3.284 (2)	134
C17—H17*A*⋯*Cg*1^i^	0.96	2.83	3.648 (5)	144

Symmetry code: (i) 
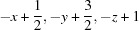
.

**Fig. 1.**
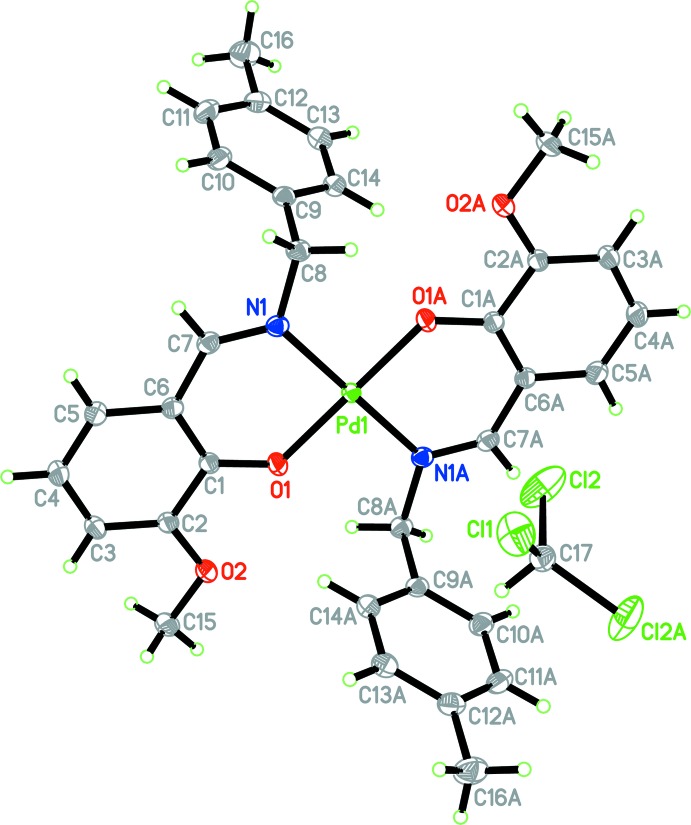
The molecular structure of (I), showing 40% probability displacement ellipsoids and the atom-numbering scheme. Atoms with suffix A of the Schiff base ligand were generated by symmetry code: 1/2 - x, 3/2 - y, 1 - z. Only one disorder component of the disordered C–H group of the chloroform solvate is shown for clarity, and Cl2A atom was generated by symmetry code: -x, y, 1/2-z.

**Fig. 2.**
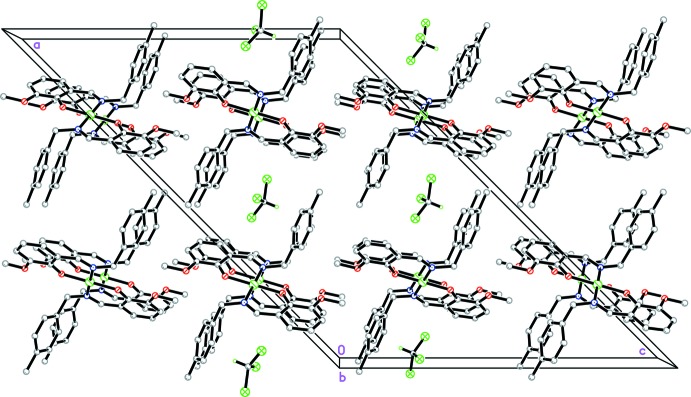
The crystal packing of (I) viewed along the b axis showing the stacking of molecules of the PdII complex. H atoms were omitted and only one disorder component of the disordered C–H group of the chloroform solvate is shown for clarity.

## supplementary crystallographic information

### S1. Comment

Complexes of palladium(II) and nickel(II) have broad and diversified applications involving numerous fields of catalysis such as the Heck reaction, Suzuki-Miyaura coupling reactions and including also the polymerization of ethylene, epoxidation and allylic alkylation (Bowes *et al.*, 2011; Gupta & Sutar, 2008; Mohd Tajuddin *et al.*, 2012; Tamizh & Karvembu, 2012). They are also important in various aspects of bioinorganic chemistry (Geeta *et al.*, 2010; Kalita *et al.*, 2014;). The properties of such complexes depend on the coordination environment around the metal center. Schiff bases containing iminoalkylphenolato groups commonly adopt a bidentate coordination mode with metal centres as for example in bis{2-[(*E*)-(4-fluorobenzyl)iminomethyl]- 6-methoxy-phenolato-*K*^2^*N*,*O*^1^}nickel(II) (Bahron *et al.*, 2011*b*). In the title complex (I), [Pd(C_32_H_32_N_2_O_4_)]·(CHCl_3_), the Schiff base ligand is bis-bidentate (see Fig. 1) and is related to the previously reported bis(2-(1-benzyliminoethyl)phenolato)palladium(II) (Bahron *et al.*, 2011*a*) but with different substituents on the iminoalkylphenolato and benzyl ring systems. Herein the crystal structure of (I) is reported.

The asymmetric unit of (I) consists of one half each of the complex molecule and the chloroform solvate molecule. The Pd^II^ atom lies on an inversion center while the Cl1 atom of the CHCl_3_ solvate lies on a two-fold axis. The C17–H17A group is disordered with equal occupancies about this axis with Cl2 in a general position with full occupancy. These two symmetry elements generate the other halves of the Schiff base ligand and the chloroform molecule. The Pd^II^ ion is four-coordinate and adopts a square planar geometry *via* cordination to the two imine N (N1 and N1^i^ symmetry code; i = 1/2 - *x*, 3/2 - *y*, 1 - *z*) and two phenolic O (O1 and O1^i^ symmetry code; i = 1/2 - *x*, 3/2 - *y*, 1 - *z*) atoms of the two bidentate Schiff base anions. The imine N atoms and phenolic O atoms are in mutually *trans* positions. The Pd—N and Pd—O distances in the N_2_O_2_ coordination [1.9741 (10) Å and 2.0204 (12) Å, respectively] are in the same ranges as those observed in the other closely related Pd^II^ complexes of N_2_O_2_ Schiff base ligands (Bahron *et al.*, 2011*a* and Halder *et al.*, 2008). Other bond lengths and angles observed in the structure are also normal (Allen *et al.*, 1987). The bond angles O–Pd–N [O1–Pd1–N1 = 92.17 (5)° and O1—Pd1–N1^i^ = 87.83 (5)° symmetry code; i = 1/2 - *x*, 3/2 - *y*, 1 - *z*] are close to 90°. Moreover the coordination of the two NO bidentate chelate ligands to the Pd^II^ ion results in the formation of two six-membered rings (Pd1/N1/C7/C8/C1/O1 and Pd1/N1^i^/C7^i^/C8^i^/C1^i^/O1^i^). The methoxy substituent deviates only slightly from the plane of the ring to which it is bound with the torsion angle C15–O2–C2–C3 = 7.9 (2)°. The benzene ring (C9–C14) makes a dihedral angle of 73.52 (10)° with the methoxyphenolate ring.

In the crystal packing (Fig. 2), molecules of the Pd^II^ complex are arranged into sheets parallel to the *ac* plane, and the chloroform solvent molecules are located in the interstitial areas between the complex molecules. Weak intermolecular C—H···O interactions stabilise the packing. A C—H···π interaction involving the centroid of the (C9–C14) benzene ring, *Cg*_1_, is also observed, (Table 1).

### S2. Experimental

The ligand, (*E*)-2-methoxy-6-((4-methylbenzylimino)methyl)-phenol (5 mmol, 1.2765 g) was dissolved in CH_3_CN (10 ml) in a round-bottomed flask. Palladium(II) acetate (2.5 mmol, 0.5612 g) was dissolved separately in CH_3_CN (10 ml) and added to the flask containing the ligand solution. The mixture was refluxed with stirring for 4 h upon which a dark yellow solid was formed. The solid was filtered off, washed with ice-cold CH_3_CN and air dried at room temperature. The solid product was recrystallized from CHCl_3_ yielding orange crystals. Yield 94.4%. Melting point 236–238 °C. ^1^H NMR (300 MHz, CDCl_3_, p.p.m.): δ = 2.30 (s, 3H, CH_3_), 5.07 (s, 2H, CH_2_), 3.75 (s, 3H, Ar-OCH_3_), 6.76–7.34 (m, 7H, ArH), 7.69 (s, 1H, =CH). ^13^C NMR (300 MHz, CDCl_3_): 21.1 (CH_3_), 55.9 (Ar-OCH_3_), 62.25 (CH_2_), 114.0, 120.4, 125.4, 128.4, 129.1, 136.1 (ArC), 162.6 (N=CH). Analytical calculation for C_32_H_32_N_2_O_4_Pd: C, 62.49; H, 5.24; N, 4.55; Found: C, 62.47; H, 5.29; N, 4.55. IR (KBr, cm^-1^): ν(C=N) 1623 (*s*), ν(C—N) 1316 (*s*), ν(C—O) 1239 (*s*), ν(OCH_3_) 1092 (w), ν(Pd—O) 660 (w), ν(Pd—N) 416 (w).

### S3. Refinement

All H atoms were positioned geometrically and allowed to ride on their parent atoms, with d(C—H) = 0.93 Å for aromatic, 0.97 Å for CH and CH_2_ and 0.96 for CH_3_ atoms. The *U*_iso_ values were constrained to be 1.5*U*_eq_ of the carrier atom for methyl H atoms and 1.2*U*_eq_ for the remaining H atoms. A rotating group model was used for the methyl groups. The highest residual electron density peak is located at 0.18 Å from Cl2 and the deepest hole is located at 0.71 Å from Cl2.

### Crystal data

**Table:**

[Pd(C_16_H_16_NO)_2_]·CHCl_3_	*F*(000) = 1496
*M**_r_* = 734.36	*D*_x_ = 1.595 Mg m^−^^3^
Monoclinic, *C*2/*c*	Melting point = 509–511 K
Hall symbol: -C 2yc	Mo *K*α radiation, λ = 0.71073 Å
*a* = 31.9861 (8) Å	Cell parameters from 5542 reflections
*b* = 5.9668 (2) Å	θ = 1.8–32.5°
*c* = 22.6135 (5) Å	µ = 0.91 mm^−^^1^
β = 134.885 (1)°	*T* = 100 K
*V* = 3057.92 (15) Å^3^	Block, orange
*Z* = 4	0.48 × 0.25 × 0.18 mm

### Data collection

**Table:**

Bruker APEXII CCD area-detector diffractometer	5542 independent reflections
Radiation source: sealed tube	5006 reflections with *I* > 2σ(*I*)
Graphite monochromator	*R*_int_ = 0.021
φ and ω scans	θ_max_ = 32.5°, θ_min_ = 1.8°
Absorption correction: multi-scan (*SADABS*; Bruker, 2009)	*h* = −48→48
*T*_min_ = 0.669, *T*_max_ = 0.853	*k* = −9→8
43800 measured reflections	*l* = −33→34

### Refinement

**Table:**

Refinement on *F*^2^	Primary atom site location: structure-invariant direct methods
Least-squares matrix: full	Secondary atom site location: difference Fourier map
*R*[*F*^2^ > 2σ(*F*^2^)] = 0.029	Hydrogen site location: inferred from neighbouring sites
*wR*(*F*^2^) = 0.073	H-atom parameters constrained
*S* = 1.05	*w* = 1/[σ^2^(*F*_o_^2^) + (0.029*P*)^2^ + 8.064*P*] where *P* = (*F*_o_^2^ + 2*F*_c_^2^)/3
5542 reflections	(Δ/σ)_max_ = 0.001
204 parameters	Δρ_max_ = 1.24 e Å^−^^3^
0 restraints	Δρ_min_ = −1.90 e Å^−^^3^

### Special details

**Table:**

Experimental. The crystal was placed in the cold stream of an Oxford Cryosystems Cobra open-flow nitrogen cryostat (Cosier & Glazer, 1986) operating at 100.0 (1) K.
Geometry. All e.s.d.'s (except the e.s.d. in the dihedral angle between two l.s. planes) are estimated using the full covariance matrix. The cell e.s.d.'s are taken into account individually in the estimation of e.s.d.'s in distances, angles and torsion angles; correlations between e.s.d.'s in cell parameters are only used when they are defined by crystal symmetry. An approximate (isotropic) treatment of cell e.s.d.'s is used for estimating e.s.d.'s involving l.s. planes.
Refinement. Refinement of *F*^2^ against ALL reflections. The weighted *R*-factor *wR* and goodness of fit *S* are based on *F*^2^, conventional *R*-factors *R* are based on *F*, with *F* set to zero for negative *F*^2^. The threshold expression of *F*^2^ > σ(*F*^2^) is used only for calculating *R*-factors(gt) *etc*. and is not relevant to the choice of reflections for refinement. *R*-factors based on *F*^2^ are statistically about twice as large as those based on *F*, and *R*- factors based on ALL data will be even larger.

### Fractional atomic coordinates and isotropic or equivalent isotropic displacement parameters (Å^2^)

**Table:**

	*x*	*y*	*z*	*U*_iso_*/*U*_eq_	Occ. (<1)
Pd1	0.2500	0.7500	0.5000	0.01355 (4)	
O1	0.27424 (5)	0.77360 (19)	0.44058 (7)	0.0199 (2)	
O2	0.29036 (5)	0.7333 (2)	0.34385 (7)	0.0216 (2)	
N1	0.29650 (5)	1.0260 (2)	0.56892 (7)	0.0161 (2)	
C1	0.30562 (6)	0.9327 (2)	0.44764 (8)	0.0163 (2)	
C2	0.31517 (6)	0.9175 (3)	0.39460 (9)	0.0182 (2)	
C3	0.34628 (7)	1.0811 (3)	0.39600 (9)	0.0230 (3)	
H3A	0.3517	1.0693	0.3607	0.028*	
C4	0.36987 (8)	1.2650 (3)	0.44999 (10)	0.0256 (3)	
H4A	0.3909	1.3743	0.4505	0.031*	
C5	0.36196 (7)	1.2838 (3)	0.50202 (10)	0.0229 (3)	
H5A	0.3777	1.4061	0.5378	0.028*	
C6	0.32999 (6)	1.1187 (2)	0.50177 (8)	0.0172 (2)	
C7	0.32398 (6)	1.1514 (2)	0.55844 (9)	0.0176 (2)	
H7A	0.3421	1.2786	0.5918	0.021*	
C8	0.30159 (6)	1.0981 (3)	0.63693 (9)	0.0180 (2)	
H8A	0.2676	1.0451	0.6249	0.022*	
H8B	0.3020	1.2605	0.6393	0.022*	
C9	0.35728 (6)	1.0068 (2)	0.72040 (9)	0.0169 (2)	
C10	0.40868 (7)	1.1352 (3)	0.77237 (9)	0.0215 (3)	
H10A	0.4083	1.2778	0.7555	0.026*	
C11	0.46054 (7)	1.0533 (3)	0.84920 (10)	0.0245 (3)	
H11A	0.4943	1.1418	0.8829	0.029*	
C12	0.46238 (7)	0.8403 (3)	0.87610 (9)	0.0220 (3)	
C13	0.41100 (7)	0.7121 (3)	0.82397 (10)	0.0213 (3)	
H13A	0.4114	0.5695	0.8409	0.026*	
C14	0.35894 (7)	0.7929 (3)	0.74686 (9)	0.0197 (3)	
H14A	0.3253	0.7038	0.7130	0.024*	
C15	0.30447 (7)	0.6982 (3)	0.29710 (10)	0.0257 (3)	
H15A	0.2903	0.5538	0.2708	0.039*	
H15B	0.3465	0.7047	0.3339	0.039*	
H15C	0.2861	0.8125	0.2552	0.039*	
C16	0.51830 (8)	0.7502 (3)	0.95912 (11)	0.0325 (4)	
H16A	0.5514	0.7914	0.9680	0.049*	
H16B	0.5160	0.5899	0.9593	0.049*	
H16C	0.5232	0.8119	1.0029	0.049*	
C17	0.00983 (14)	0.8371 (6)	0.2433 (2)	0.0230 (6)	0.50
H17A	0.0259	0.8362	0.2200	0.028*	0.50
Cl1	0.0000	1.12294 (11)	0.2500	0.04186 (16)	
Cl2	0.05936 (2)	0.70272 (10)	0.33308 (4)	0.04878 (16)	

### Atomic displacement parameters (Å^2^)

**Table:**

	*U*^11^	*U*^22^	*U*^33^	*U*^12^	*U*^13^	*U*^23^
Pd1	0.01277 (6)	0.01580 (7)	0.01323 (7)	−0.00043 (4)	0.00959 (6)	−0.00051 (4)
O1	0.0230 (5)	0.0231 (5)	0.0223 (5)	−0.0064 (4)	0.0191 (5)	−0.0050 (4)
O2	0.0230 (5)	0.0280 (6)	0.0217 (5)	−0.0055 (4)	0.0185 (5)	−0.0047 (4)
N1	0.0145 (5)	0.0181 (5)	0.0143 (5)	0.0009 (4)	0.0097 (4)	−0.0003 (4)
C1	0.0133 (5)	0.0203 (6)	0.0138 (5)	0.0002 (4)	0.0090 (5)	0.0020 (4)
C2	0.0144 (5)	0.0243 (7)	0.0141 (5)	−0.0012 (5)	0.0095 (5)	0.0014 (5)
C3	0.0205 (6)	0.0314 (8)	0.0182 (6)	−0.0042 (6)	0.0141 (6)	0.0022 (6)
C4	0.0247 (7)	0.0297 (8)	0.0210 (7)	−0.0081 (6)	0.0156 (6)	0.0016 (6)
C5	0.0213 (7)	0.0251 (7)	0.0179 (6)	−0.0064 (5)	0.0122 (6)	−0.0005 (5)
C6	0.0144 (5)	0.0200 (6)	0.0132 (5)	−0.0006 (5)	0.0083 (5)	0.0019 (5)
C7	0.0152 (5)	0.0179 (6)	0.0147 (5)	−0.0001 (5)	0.0088 (5)	0.0001 (4)
C8	0.0182 (6)	0.0192 (6)	0.0181 (6)	0.0007 (5)	0.0134 (5)	−0.0023 (5)
C9	0.0177 (6)	0.0191 (6)	0.0159 (5)	−0.0001 (5)	0.0126 (5)	−0.0029 (5)
C10	0.0219 (6)	0.0203 (6)	0.0191 (6)	−0.0032 (5)	0.0134 (6)	−0.0033 (5)
C11	0.0206 (6)	0.0264 (7)	0.0186 (6)	−0.0046 (6)	0.0111 (6)	−0.0043 (5)
C12	0.0207 (6)	0.0271 (7)	0.0173 (6)	0.0025 (5)	0.0131 (6)	−0.0005 (5)
C13	0.0235 (7)	0.0220 (6)	0.0213 (6)	0.0017 (5)	0.0168 (6)	0.0008 (5)
C14	0.0197 (6)	0.0223 (6)	0.0192 (6)	−0.0014 (5)	0.0145 (6)	−0.0016 (5)
C15	0.0249 (7)	0.0374 (9)	0.0234 (7)	−0.0029 (6)	0.0202 (6)	−0.0036 (6)
C16	0.0251 (8)	0.0385 (10)	0.0215 (7)	0.0054 (7)	0.0120 (7)	0.0050 (7)
C17	0.0241 (14)	0.0232 (14)	0.0245 (14)	−0.0037 (11)	0.0181 (12)	−0.0041 (11)
Cl1	0.0565 (4)	0.0199 (3)	0.0593 (5)	0.000	0.0445 (4)	0.000
Cl2	0.0256 (2)	0.0353 (2)	0.0438 (3)	0.00892 (18)	0.0097 (2)	−0.0081 (2)

### Geometric parameters (Å, º)

**Table:**

Pd1—O1^i^	1.9741 (10)	C9—C14	1.395 (2)
Pd1—O1	1.9741 (10)	C10—C11	1.393 (2)
Pd1—N1	2.0204 (12)	C10—H10A	0.9300
Pd1—N1^i^	2.0204 (12)	C11—C12	1.392 (2)
O1—C1	1.3069 (17)	C11—H11A	0.9300
O2—C2	1.3672 (19)	C12—C13	1.393 (2)
O2—C15	1.4279 (18)	C12—C16	1.508 (2)
N1—C7	1.2971 (19)	C13—C14	1.396 (2)
N1—C8	1.4911 (18)	C13—H13A	0.9300
C1—C6	1.411 (2)	C14—H14A	0.9300
C1—C2	1.4344 (19)	C15—H15A	0.9600
C2—C3	1.378 (2)	C15—H15B	0.9600
C3—C4	1.401 (2)	C15—H15C	0.9600
C3—H3A	0.9300	C16—H16A	0.9600
C4—C5	1.372 (2)	C16—H16B	0.9600
C4—H4A	0.9300	C16—H16C	0.9600
C5—C6	1.417 (2)	C17—C17^ii^	0.871 (6)
C5—H5A	0.9300	C17—Cl2	1.654 (3)
C6—C7	1.437 (2)	C17—Cl1	1.760 (3)
C7—H7A	0.9300	C17—Cl2^ii^	1.769 (3)
C8—C9	1.512 (2)	C17—H17A	0.9604
C8—H8A	0.9700	Cl1—C17^ii^	1.760 (3)
C8—H8B	0.9700	Cl2—C17^ii^	1.769 (3)
C9—C10	1.394 (2)		
			
O1^i^—Pd1—O1	180.000 (1)	C10—C9—C8	120.25 (14)
O1^i^—Pd1—N1	87.83 (5)	C14—C9—C8	121.32 (13)
O1—Pd1—N1	92.17 (5)	C11—C10—C9	121.09 (15)
O1^i^—Pd1—N1^i^	92.17 (5)	C11—C10—H10A	119.5
O1—Pd1—N1^i^	87.83 (5)	C9—C10—H10A	119.5
N1—Pd1—N1^i^	180.0	C12—C11—C10	120.73 (15)
C1—O1—Pd1	127.25 (9)	C12—C11—H11A	119.6
C2—O2—C15	116.10 (12)	C10—C11—H11A	119.6
C7—N1—C8	115.30 (12)	C11—C12—C13	118.08 (14)
C7—N1—Pd1	123.66 (10)	C11—C12—C16	121.11 (16)
C8—N1—Pd1	121.04 (9)	C13—C12—C16	120.82 (16)
O1—C1—C6	125.72 (13)	C12—C13—C14	121.50 (15)
O1—C1—C2	116.75 (13)	C12—C13—H13A	119.2
C6—C1—C2	117.52 (13)	C14—C13—H13A	119.2
O2—C2—C3	124.80 (13)	C9—C14—C13	120.18 (14)
O2—C2—C1	114.39 (12)	C9—C14—H14A	119.9
C3—C2—C1	120.81 (14)	C13—C14—H14A	119.9
C2—C3—C4	120.72 (14)	O2—C15—H15A	109.5
C2—C3—H3A	119.6	O2—C15—H15B	109.5
C4—C3—H3A	119.6	H15A—C15—H15B	109.5
C5—C4—C3	119.97 (15)	O2—C15—H15C	109.5
C5—C4—H4A	120.0	H15A—C15—H15C	109.5
C3—C4—H4A	120.0	H15B—C15—H15C	109.5
C4—C5—C6	120.60 (15)	C12—C16—H16A	109.5
C4—C5—H5A	119.7	C12—C16—H16B	109.5
C6—C5—H5A	119.7	H16A—C16—H16B	109.5
C1—C6—C5	120.37 (13)	C12—C16—H16C	109.5
C1—C6—C7	122.75 (13)	H16A—C16—H16C	109.5
C5—C6—C7	116.89 (14)	H16B—C16—H16C	109.5
N1—C7—C6	128.23 (14)	C17^ii^—C17—Cl2	82.7 (4)
N1—C7—H7A	115.9	C17^ii^—C17—Cl1	75.67 (10)
C6—C7—H7A	115.9	Cl2—C17—Cl1	115.99 (18)
N1—C8—C9	111.02 (11)	C17^ii^—C17—Cl2^ii^	68.1 (4)
N1—C8—H8A	109.4	Cl2—C17—Cl2^ii^	115.65 (19)
C9—C8—H8A	109.4	Cl1—C17—Cl2^ii^	110.29 (18)
N1—C8—H8B	109.4	C17^ii^—C17—H17A	171.5
C9—C8—H8B	109.4	Cl2—C17—H17A	104.5
H8A—C8—H8B	108.0	Cl1—C17—H17A	104.5
C10—C9—C14	118.42 (14)	Cl2^ii^—C17—H17A	104.4
			
N1—Pd1—O1—C1	−4.24 (13)	C4—C5—C6—C7	179.83 (15)
N1^i^—Pd1—O1—C1	175.76 (13)	C8—N1—C7—C6	176.22 (13)
O1^i^—Pd1—N1—C7	−175.14 (12)	Pd1—N1—C7—C6	−3.4 (2)
O1—Pd1—N1—C7	4.86 (12)	C1—C6—C7—N1	−0.7 (2)
O1^i^—Pd1—N1—C8	5.26 (10)	C5—C6—C7—N1	179.23 (15)
O1—Pd1—N1—C8	−174.74 (10)	C7—N1—C8—C9	−85.20 (15)
Pd1—O1—C1—C6	1.8 (2)	Pd1—N1—C8—C9	94.43 (13)
Pd1—O1—C1—C2	−177.17 (10)	N1—C8—C9—C10	93.72 (16)
C15—O2—C2—C3	7.9 (2)	N1—C8—C9—C14	−85.35 (16)
C15—O2—C2—C1	−173.07 (13)	C14—C9—C10—C11	−0.4 (2)
O1—C1—C2—O2	−0.92 (19)	C8—C9—C10—C11	−179.47 (14)
C6—C1—C2—O2	180.00 (12)	C9—C10—C11—C12	0.1 (2)
O1—C1—C2—C3	178.12 (14)	C10—C11—C12—C13	0.1 (2)
C6—C1—C2—C3	−1.0 (2)	C10—C11—C12—C16	179.97 (16)
O2—C2—C3—C4	179.63 (15)	C11—C12—C13—C14	0.0 (2)
C1—C2—C3—C4	0.7 (2)	C16—C12—C13—C14	−179.83 (15)
C2—C3—C4—C5	−0.2 (3)	C10—C9—C14—C13	0.5 (2)
C3—C4—C5—C6	−0.1 (3)	C8—C9—C14—C13	179.60 (13)
O1—C1—C6—C5	−178.26 (14)	C12—C13—C14—C9	−0.3 (2)
C2—C1—C6—C5	0.7 (2)	Cl2—C17—Cl1—C17^ii^	−74.3 (4)
O1—C1—C6—C7	1.7 (2)	Cl2^ii^—C17—Cl1—C17^ii^	59.6 (4)
C2—C1—C6—C7	−179.34 (13)	Cl1—C17—Cl2—C17^ii^	70.1 (2)
C4—C5—C6—C1	−0.2 (2)	Cl2^ii^—C17—Cl2—C17^ii^	−61.4 (3)

Symmetry codes: (i) −*x*+1/2, −*y*+3/2, −*z*+1; (ii) −*x*, *y*, −*z*+1/2.

### Hydrogen-bond geometry (Å, º)

*Cg*1 is the centroid of the C9–C14 ring.

**Table:**

*D*—H···*A*	*D*—H	H···*A*	*D*···*A*	*D*—H···*A*
C8—H8*A*···O1^i^	0.97	2.19	2.806 (2)	120
C14—H14*A*···O1^i^	0.93	2.57	3.284 (2)	134
C17—H17*A*···*Cg*1^i^	0.96	2.83	3.648 (5)	144

Symmetry code: (i) −*x*+1/2, −*y*+3/2, −*z*+1.
